# Complete Genome Sequence of Aeromonas hydrophila Bacteriophage BUCT552

**DOI:** 10.1128/mra.01172-21

**Published:** 2022-02-17

**Authors:** Rujia Chen, Xuling Xu, Pengjun Han, Yunjia Hu, Hongbo Qin, Mengzhe Li, Xiaoping An, Lihua Song, Huahao Fan, Yigang Tong

**Affiliations:** a College of Life Science and Technology, Beijing University of Chemical Technology, Beijing, China; b Beijing Advanced Innovation Center for Soft Matter Science and Engineering (BAIC-SM), Beijing University of Chemical Technology, Beijing, China; c Phagelux (Nanjing) Bio-tech Co., Ltd., Nanjing, China; DOE Joint Genome Institute

## Abstract

We report the complete genome sequence of Aeromonas hydrophila bacteriophage BUCT552 whose full length of the linear dsDNA genome is 59,685 bp and G+C content is 60.0%. It contains 74 open reading frames but no tRNA. The results of TEM showed BUCT552 is a member of the family *Siphoviridae*.

## ANNOUNCEMENT

Aeromonas hydrophila belongs to *Vibrionaceae*, a family of Gram-negative bacteria (1, 2) and can be found in different aquatic environments, foods, and food processing systems (3). It is an emerging bacterial pathogen, and not only a thorny problem in the aquaculture industry, but also a severe threat to human life and health (4). Bacteriophages, as a very potential antibiotic substitute, have shown good application prospects in the prevention and treatment of human and animal diseases (5–8).

In this research, A. hydrophila bacteriophage BUCT552 was isolated from the sewage of Zhongcai Aquatic Products Wholesale Market in Nanjing City, Jiangsu Province, China (118.7674° E, 32.0415° N) in August 2020. A. hydrophila host strains were cultivated in tryptic soy broth (TSB) at 29°C, 200 r/min for 8 h. Individual phages were isolated using the enrichment method, then purified with three rounds of plaque assays on double-layer tryptic soy agar (TSA) plates at 29°C overnight, obtaining clear single plaques. The phage morphology was observed by uranyl acetate negative-stain method (9). Phage genomic DNA was extracted using the phenol-chloroform method (10). The Illumina MiSeq sequencer was used to sequence the whole genome of the phage following the NEBNext Ultra II DNA library prep kit for Illumina (NEB, E7805) to construct paired-end 2 × 150-bp reads sequencing library. The genome sequence was assembled using SPAdes v3.13.0 (11). RAST (http://rast.nmpdr.org/) online annotation was used to quickly annotate the whole genome, and then the online NCBI BLASTp tool (http://www.ncbi.nlm.nih.gov/BLAST) was used to confirm RAST annotations (12). Then, tRNAscan-SE v2.0 was used (http://lowelab.ucsc.edu/cgi-bin/tRNAscan-SE2.cgi) (13) to predict tRNA in the genome. All tools were run with default parameters unless otherwise specified.

**FIG. 1:**
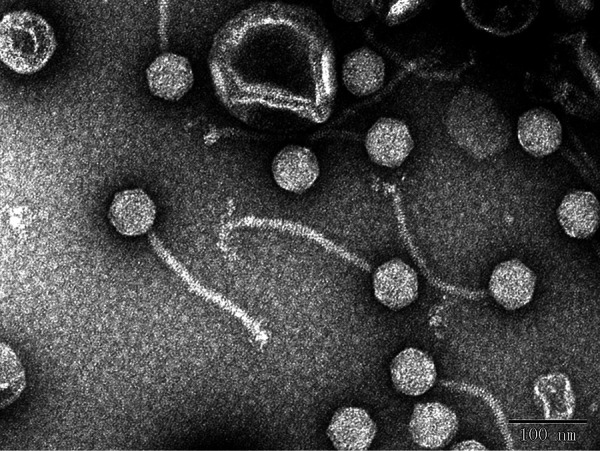
The morphology of phage BUCT552 was examined with a transmission electron microscope (JEM-1200EX, Japan) at 80 kV. Four virions were measured in the figure.

Transmission electron micrograph of BUCT552 showed that the capsid was a regular icosahedral body with a diameter of 55 ± 3 nm and a tail length of 200 ± 3 nm (Fig. 1). According to traditional morphology-based classification, BUCT552 belongs to the family *Siphoviridae*. The genome phage BUCT552 assembled as a circular contig of length 59,685 bp (repeats are typically omitted when reporting the length of circular contigs), and the GC content is 60.0%. The average coverage depth of the genome is 190.0. The results of RAST gene annotation showed a total of 74 open reading frames (ORF), and no tRNA was predicted in the genome. There are 24 predicted ORFs which encode structure, packaging, replication, modification, and lysis genes, thus the gene density is 92.5%. Its genome was compared with other known phages in BLASTn, showing that the three closest phages are Aeromonas phage LAh_7, vB_AhyS-A18P4, BUCT551, and the query cover was 3%, 8%, and 3% with the corresponding identity 75.59%, 76.30%, and 75.22%, respectively.

In this study, a lytic phage BUCT552 which can infect and lyse A. hydrophila was isolated from sewage. The phage displays a maximum of 8% similarity with known phages, and it enriches our understanding about the diversity of A. hydrophila phages.

### Data availability.

The accession number of whole genome sequence of phage BUCT552 in the GenBank database is MW978786. The accession number of the raw reads in NCBI SRA is SRR17399946.
